# Non-additive QTL mapping of lactation traits in 124,000 cattle reveals novel recessive loci

**DOI:** 10.1186/s12711-021-00694-3

**Published:** 2022-01-24

**Authors:** Edwardo G. M. Reynolds, Thomas Lopdell, Yu Wang, Kathryn M. Tiplady, Chad S. Harland, Thomas J. J. Johnson, Catherine Neeley, Katie Carnie, Richard G. Sherlock, Christine Couldrey, Stephen R. Davis, Bevin L. Harris, Richard J. Spelman, Dorian J. Garrick, Mathew D. Littlejohn

**Affiliations:** 1 Massey University, Palmerston North, New Zealand; 2 Livestock Improvement Corporation, Hamilton, New Zealand

## Abstract

**Background:**

Deleterious recessive conditions have been primarily studied in the context of Mendelian diseases. Recently, several deleterious recessive mutations with large effects were discovered via non-additive genome-wide association studies (GWAS) of quantitative growth and developmental traits in cattle, which showed that quantitative traits can be used as proxies of genetic disorders when such traits are indicative of whole-animal health status. We reasoned that lactation traits in cattle might also reflect genetic disorders, given the increased energy demands of lactation and the substantial stresses imposed on the animal. In this study, we screened more than 124,000 cows for recessive effects based on lactation traits.

**Results:**

We discovered five novel quantitative trait loci (QTL) that are associated with large recessive impacts on three milk yield traits, with these loci presenting missense variants in the *DOCK8*, *IL4R*, *KIAA0556*, and *SLC25A4* genes or premature stop variants in the *ITGAL*, *LRCH4*, and *RBM34* genes, as candidate causal mutations. For two milk composition traits, we identified several previously reported additive QTL that display small dominance effects. By contrasting results from milk yield and milk composition phenotypes, we note differing genetic architectures. Compared to milk composition phenotypes, milk yield phenotypes had lower heritabilities and were associated with fewer additive QTL but had a higher non-additive genetic variance and were associated with a higher proportion of loci exhibiting dominance.

**Conclusions:**

We identified large-effect recessive QTL which are segregating at surprisingly high frequencies in cattle. We speculate that the differences in genetic architecture between milk yield and milk composition phenotypes derive from underlying dissimilarities in the cellular and molecular representation of these traits, with yield phenotypes acting as a better proxy of underlying biological disorders through presentation of a larger number of major recessive impacts.

**Supplementary Information:**

The online version contains supplementary material available at 10.1186/s12711-021-00694-3.

## Background

Non-additive genetic effects are best known from studies of Mendelian diseases, where recessive conditions have been shown to have major deleterious impacts on health and performance. These studies have mostly used a ‘forward genetics’ approach, where the observation of a disease phenotype precedes fine mapping and sequencing to highlight the mutation [1–3]. However, the reverse approach has also been applied, which first identifies candidate loss-of-function genotypes and subsequently performs phenotyping on traits likely to reflect the impact of the mutation [4–6]. Genome-wide association studies (GWAS) have been used to investigate non-additive effects in quantitative traits, but the number of findings remains limited in comparison to additive effects, where most such analyses fit an additive model only. Recent studies of non-additive effects include the investigation of complex traits in both humans [7] and cattle [8–12]. In cattle, Reynolds et al*.* [12] identified several recessive mutations with major negative impacts on growth and developmental traits, where some of these effects were found to be due to underlying genetic syndromes.

The concept of using routinely gathered, quantitative traits as proxies of genetic disorders is based on the idea that phenotypes such as growth or liveweight might be indicative of the overall health status of the animal, e.g. reduced growth could be caused by an underlying genetic disorder, in which case such effects could be detected via GWAS. Thus, it is relevant to investigate whether other easily measured traits might also serve as proxies of animal fitness, with a view to extend the scope of this approach. Lactation traits such as milk volume comprise one of the most commonly targeted classes of quantitative traits studied in cattle, where additive analyses of these traits have identified numerous candidate causative genes such as *DGAT1* [13], *GHR* [14], *ABCG2* [15], *GPAT4* [16], and *MGST1* [17]. Lactation traits might also reflect genetic disorders, given the increased energy demands of lactation and the substantial metabolic and physiological stresses imposed on the animal [18]. Thus, we were interested in investigating whether the application of non-additive models to lactation data might identify recessive mutations in addition to those found for growth traits, and to this end, have conducted non-additive GWAS for milk traits on 124,000 animals. We contrast the additive and non-additive genetic architectures of milk yield traits and milk composition traits. Finally, we describe the discovery of several novel major effect recessive loci and highlight candidate mutations that could underlie these undiagnosed recessive disorders.

## Methods

### Animal populations

The dataset reported in this study consists of 124,364 New Zealand dairy cattle. These animals come from a mixed breed population, where 20,893 were recorded as 16/16^th^’s Holstein–Friesian (HF), 13,184 were recorded as 16/16^th^’s Jersey (J), 67,520 were crosses with varying proportions of the two breeds (HFXJ), and 22,767 were HF or J crossbreeds with minor proportions of other breeds including Ayrshire, Brown Swiss, or Hereford (and other crosses). The breed of an individual may be coded as 16/16^th^s, however, this does not preclude the possibility that an ancestor may have been crossbred since matings between 15/16^th^s and 16/16^th^s animals are recorded as producing 16/16^th^s offspring. The animals were born between 1990 and 2018 with a mean birth year of 2010.

### Phenotypes

We analysed five first-lactation yield deviation phenotypes: three milk yield traits, i.e. milk volume (L/lactation; a lactation refers to a standardised 268-day lactation period; N = 124,356), milk protein yield (kg/lactation; N = 124,356), and milk fat yield (kg/lactation; N = 124,356); and two milk composition traits, i.e. milk protein percentage (%; N = 124,363), and milk fat percentage (%; N = 124,363). Milk protein yield and milk fat yield are calculated on individual herd tests and are the product of the herd test milk volume multiplied by the herd test milk protein percentage or milk fat percentage, respectively.

Prior to genetic analysis, the phenotypes were adjusted for non-genetic effects obtained from the national genetic evaluation of the entire cattle population (30 $\times$ 10^6^ animals), which fits a mixed linear model, including effects for: contemporary group, age at calving, stage of lactation, and record type (i.e. am milkings, or pm milkings, or both). Since the number of herd-test measurements varies for each animal, these adjusted test day phenotypes were aggregated to a first lactation phenotypic deviation such that each animal has a single record and a corresponding weighting that reflects the amount of information contributing to the record [19].

### Reference population for sequence-based imputation

Whole-genome sequencing was performed on 1300 animals that were mostly ancestral sires and represented the reference population for sequence-based imputation. These animals: HF (N = 306), J (N = 219), HFXJ (N = 717), or other breeds and crossbreeds (N = 58); were sequenced on Illumina HiSeq 2000 instruments targeting 100-bp paired-end reads. The sequence data were aligned to the ARS-UCD1.2 reference genome assembly using the Burrows–Wheeler alignment algorithm (BWA) version 0.7.17 [20], which resulted in a mean read depth of 15$\times$. For variant calling, we used the Genome Analysis ToolKit (GATK) v4.0.6.0 [21], followed by filtering of the variants with the variant quality score recalibration technique [21]. Based on the animals with a read depth > 10$\times$ (N = 850), variants that were singletons or were multi-allelic, had a map quality score lower than 50, or a Mendelian error rate higher than 5%, were filtered out leaving 21,005,869 whole-genome sequence variants. The genotypes at the positions of these filtered variants were extracted from the sequence data of all 1300 animals and were phased using the software Beagle 5.0 [22] to generate the sequence-based imputation reference panel.

### Genotyping

DNA was extracted either from ear-punch tissue samples or blood samples for the 124,364 animals included in our study. These samples were processed to extract DNA at GeneMark (Hamilton, New Zealand) using Qiagen BioSprint kits, or at GeneSeek (Lincoln, NE, USA) using the Life Technologies’ MagMAX system. Genotyping was performed using a variety of single nucleotide polymorphism (SNP) arrays including GeneSeek GGPv1 (8729 SNPs), GGPv2 (20,012 SNPs), GGPv2.1 (20,015 SNPs), GGPv3 (31,813 SNPs), GGPv3.1 (31,945 SNPs), GGPv4 (37,092 SNPs), GGP50kv1 (48,156 SNPs), GGP50kv1.1 (48,161 SNPs), Illumina BovineSNP50v1 (53,126 SNPs), Illumina BovineSNP50v2 (53,629 SNPs), or the BovineHD (772,235 SNPs) chips.

### Consolidation of SNP-chip panels for sequence imputation

Imputation from the genotyping panels to sequence resolution was performed as described in Wang et al*.* [23]. The various genotyping panels were grouped into four sets: GGP panels (GGPv1, GGPv2, GGPv2.1, GGPv3, GGPv3.1, and GGPv4), 50K panels (BovineSNP50v1 and BovineSNP50v2), GGP50k panels (GGP50kv1 and GGP50kv1.1), and the BovineHD panel. Animals genotyped on the GGP panels were imputed to the BovineSNP50v1 panel, then combined with the physically genotyped 50K panel animals and successively imputed to the BovineHD panel. Animals genotyped on the GGP50k panels were separately imputed to the BovineHD panel in a single step. In order to incorporate the custom content that had been genotyped on the GGPv3 platform, we conducted similar imputation steps to impute all animals to GGPv3. Then, we combined the imputed and physically genotyped panels (imputed HD, imputed GGPv3, and physically genotyped HD), and finally imputed the resulting animals to sequence resolution using the sequence-based imputation reference population, described above. LINKPHASE3 [24] and Beagle 5.0 [22] were used for all phasing and imputation steps. In Beagle 5.0, we applied the default parameters except for effective population size that was set at 400, and a window size of 20 Mb was used except for chromosomes 7, 10, 12, 14, and 23, for which a 7-Mb window size was applied because of the greater computational demands for these chromosomes, probably due to assembly and structural complexities (as previously reported [25]). Very rare variants (homozygous alternate count ≤ 5) were removed by post-imputation filtering and poorly imputed variants based on the dosage R^2^ statistic (DR^2^; DR^2^ < 0.7) were also filtered out. In total, 16,640,294 variants remained for the GWAS and further analyses.

### Genotypes for the adjustment of population structure

We used the genotyping data from the Bovine SNP50 chip platforms to account for spurious effects due to population structure. From the initial 54,708 autosomal SNPs, markers with a high missing genotype rate (> 0.01), a low minor allele frequency (< 0.02), or that deviated from the expected Hardy–Weinberg equilibrium (> 0.15, calculated within breed) were excluded. An additional filtering step was carried out to remove poorly imputed markers (DR^2^ < 0.9) and markers in high linkage disequilibrium (LD) with another marker on the panel (pairwise R^2^ > 0.9, within 1 Mb). After these edits, a set of 31,451 SNPs remained for subsequent analyses.

### Heritability estimates

We estimated breed-specific additive and dominance heritabilities based on genomic relationship matrices (GRM) using the GCTA software [7, 26]. Additive and dominance variance components were estimated simultaneously from purebred individuals (HF = 20,893 and J = 13,184), using the same set of 31,451 filtered BovineSNP50 SNPs as for population structure adjustment (see previous section). The GCTA software estimates the variance components using a restricted maximum likelihood (REML) approach. It estimates the additive heritability (h^2^) as the ratio of additive genetic variance to phenotypic variance, and dominance heritability (δ^2^) as the ratio of dominance genetic variance to phenotypic variance. We analysed yield deviations which aggregate the herd test records that are described in the “Phenotypes” section, thus no additional records not already described were used in this analysis.

### GWAS

#### Overview of the model

We applied a non-additive GWAS approach that is similar to that described in Reynolds et al*.* [12] to identify non-additive QTL for milk traits. This approach is a two-step method that leaves-one-segment-out (LOSO) and fits all other genomic SNP effects among the 31,451 SNPs to adjust for population structure, and then applies a Markov chain Monte Carlo (MCMC) method to test the effects of all imputed-to-sequence variants in the segment that had been left out, one at a time. In general, for each sequence variant the method fits the following model:

1 $$\mathbf{y}=\bf{1}\mu +\mathbf{T}\mathbf{b}+{\mathbf{M}}_{{\varvec{\upalpha}}}{\varvec{\upalpha}}+{\mathbf{M}}_{{\varvec{\updelta}}}{\varvec{\updelta}}+ \mathbf{e},$$

where $\mathbf{y}$ is the vector of one of the five phenotypes of interest that were pre-adjusted as described in the “Phenotypes” section; $\mu$ is the overall mean; **1** is a vector of 1s; $\mathbf{b}$ is a vector of genotype class effects for the sequence variant of interest; $\mathbf{T}$ is the design matrix relating records to genotype class for the sequence variant; ${\varvec{\upalpha}}$ is a vector of random additive effects of SNPs spanning the whole genome except the segment of interest such that ${{\varvec{\upalpha}}\sim N(\bf{0}, \mathbf{I}{\sigma }}_{\alpha }^{2})$, and $\mathbf{I}$ is an identity matrix of order equal to the number of SNP effects and ${\sigma }_{\alpha }^{2}$ is the additive variance of the SNP effects; ${\varvec{\updelta}}$ is a vector of random dominance effects of SNPs spanning the whole genome except the segment of interest such that ${\varvec{\updelta}} \sim N(\bf{0}, \mathbf{I}{\sigma }_{\delta }^{2})$, and ${\sigma }_{\delta }^{2}$ is the dominance variance of the SNP effects; ${\mathbf{M}}_{{\varvec{\upalpha}}}$ and ${\mathbf{M}}_{{\varvec{\updelta}}}$ are matrices in which each column represents the covariate values for a marker locus ([0, 1, 2] and [0, 1, 0], respectively); and $\mathbf{e}$ is the vector of residual errors with $\mathbf{e} \sim N(\bf{0}, \mathbf{R})$, such that for a simple model based on single observations $\mathbf{R}=\mathbf{I}{\upsigma }_{\mathrm{e}}^{2}$, and $\mathbf{I}$ is an identity matrix of order equal to the number of phenotypic records and ${\upsigma }_{\mathrm{e}}^{2}$ is the residual error variance. Since the traits investigated here are represented by the mean of a variable number of repeated test day observations, the diagonal elements of $\mathbf{R}$ varied according to the number of observations contributing to the yield deviation. One notable contrast to the model previously implemented in Reynolds et al*.* [12], is that, here, we fit both additive (${\mathbf{M}}_{{\varvec{\upalpha}}}$) and dominance (${\mathbf{M}_{{\varvec{\updelta}}}}$) effects of the genomic markers to adjust for population structure. This modification was made to better control the inflation that was observed when analysing milk traits in a population larger than that studied in Reynolds et al. [12].

#### Adjustment of population structure

Five hundred samples of vectors of plausible additive and dominance SNP effects, $\widetilde{{\varvec{\upalpha}}}$ and $\widetilde{{\varvec{\updelta}}}$, were generated for the 31,451 SNPs using single-site Gibbs sampling based on the BayesC0 algorithm implemented in the GenSel program using standard priors [27]. The fitted model omitted the $\mathbf{T}\mathbf{b}$ term from Eq. (1) and the convergence of the Markov chain of plausible SNP effects was determined using the Geweke diagnostic [28]. The LOSO approach was used to avoid fitting effects of nearby SNPs that are in linkage disequilibrium with the sequence variant being tested. The genome was partitioned into 10-Mb LOSO intervals and, for each interval, phenotypes were adjusted for the samples of SNP effects except for those within the relevant LOSO interval. This produced distinct LOSO-adjusted phenotypic deviations for each 10-Mb interval for each sample of plausible SNP effects.

#### Association analysis

We sampled the effects of genotype classes for each sequence variant separately, for every plausible sample of LOSO-adjusted phenotypic deviations. We obtained MCMC chains of additive and dominance genotypic effects, and standard-additive effects as contrasts of these plausible effects of genotype classes. The posterior distributions were summarised in terms of their posterior means, posterior standard deviations, and z-statistics that assumed a standard normal distribution [29]. The statistical significance of standard-additive, additive, and dominance genetic effects were evaluated using a Z-test.

#### QTL identification, significance criteria, and annotation

Our primary aim was to detect non-additive QTL, thus we declared variants as significant if the dominance genotypic effect, $d$, passed a false discovery rate (FDR) threshold of 1 × 10^–3^. For each phenotype, this FDR threshold was calculated using q-values [30] as implemented in the *qvalue* package in R [31]. Since we were particularly interested in medium- to large-effect QTL, only the loci with effect sizes ($a$ or $d$) greater than 5% of the phenotypic standard deviation of the trait were considered for further downstream analyses. We calculated the dominance coefficient $k=\frac{d}{\left|a\right|}$ for each significant QTL to characterise the underlying non-additive mechanism where $k$ ≈ 0 represents a completely additive locus, $k$ ≈ 1 a completely recessive locus, $k$ < 1 a partially dominant locus, and $k$ > 1 an over-dominant locus. For standard additive effects, $\mathrm{\alpha }$, we used GCTA-COJO [32] to detect tag variants for QTL identified in our standard additive GWAS. The GCTA-COJO routine uses LD structure and GWAS summary statistics to iteratively identify significant QTL at the FDR threshold of 1 × 10^–3^.

We used sequence annotations from variant effect predictor (VEP; Ensembl 97, [33]) to highlight mutations that might be responsible for the non-additive QTL identified, and then used SIFT scores to evaluate the potential impact of any missense mutations on protein function [34]. To assess the quality of VEP-derived variant annotations and ensure that the predicted missense and nonsense variants intersected expressed exons, we manually visualised mammary RNA-seq alignments as described in Reynolds et al*.* [12] using the Integrative Genomics Viewer (IGV) [35]. These analyses confirmed that, for the three non-sense candidate mutations identified in *ITGAL*, *LRCH4*, and *RBM34*, all appeared to encode valid premature stop variants, and in the case of the *LRCH4* mutation, its position that is adjacent to the exon 3 splice acceptor boundary suggested that the variant might also have splicing consequences. We also manually inspected genome sequence alignments representing the non-additive QTL regions in animals of contrasting QTL genotyping (i.e. those carrying opposing alleles of the QTL tag SNPs), to look for possible gene-disrupting structural variants in these regions.

#### Iterative GWAS

We were interested in determining if multiple dominance QTL might segregate at associated loci, thus we implemented an iterative GWAS approach to differentiate QTL. First, we identified on each chromosome the variants with an FDR lower than the threshold. Next, we adjusted the phenotype for the effects of the genotype classes of the most significant variant (or candidate causal variant if identified) and then re-ran the GWAS model on the chromosome of interest using the adjusted phenotype. This process was iteratively repeated until no significant QTL remained on the chromosome.

## Results

### Heritabilities of lactation traits

First, we estimated the additive and dominance heritabilities for each phenotype within each breed to investigate the additive and non-additive genetic architecture of each trait. These results (Table 1) show that the dominance heritabilities were far outweighed by the additive heritabilities. This was not surprising as the values presented are of similar magnitude to those reported for other traits and populations in the literature [9, 36]. Milk fat yield in Jersey cows had the highest dominance heritability at 0.074, and milk protein percentage in Holstein–Friesian cows had the lowest dominance heritability at 0. It should be noted that there was a clear contrast between the relative heritabilities of milk composition and milk yield traits, with milk composition traits displaying high additive heritabilities but near to zero dominance heritabilities, whereas milk yield traits displayed lower additive heritabilities but higher dominance heritabilities (Table 1).

**Table 1:** Heritability estimates for lactation traits

Trait	${h}_{\mathrm{HF}}^{2}$ (SE)	${\delta }_{\mathrm{HF}}^{2}$ (SE)	${h}_{\mathrm{J}}^{2}$ (SE)	${\delta }_{\mathrm{J}}^{2}$ (SE)
Milk volume	0.296 (0.010)	0.044 (0.007)	0.312 (0.012)	0.064 (0.009)
Milk fat yield	0.261 (0.010)	0.059 (0.008)	0.232 (0.012)	0.074 (0.010)
Milk protein yield	0.235 (0.009)	0.053 (0.008)	0.236 (0.012)	0.073 (0.010)
Milk fat percentage	0.700 (0.007)	0.006 (0.004)	0.616 (0.010)	0.015 (0.006)
Milk protein percentage	0.642 (0.008)	0 (0.005)	0.636 (0.010)	0.005 (0.005)

${h}^{2}$: additive heritability: ${\delta }^{2}$: dominance heritability; HF: Holstein–Friesian, J: Jersey; SE: standard error

### GWAS for lactation traits

**Fig. 1:**
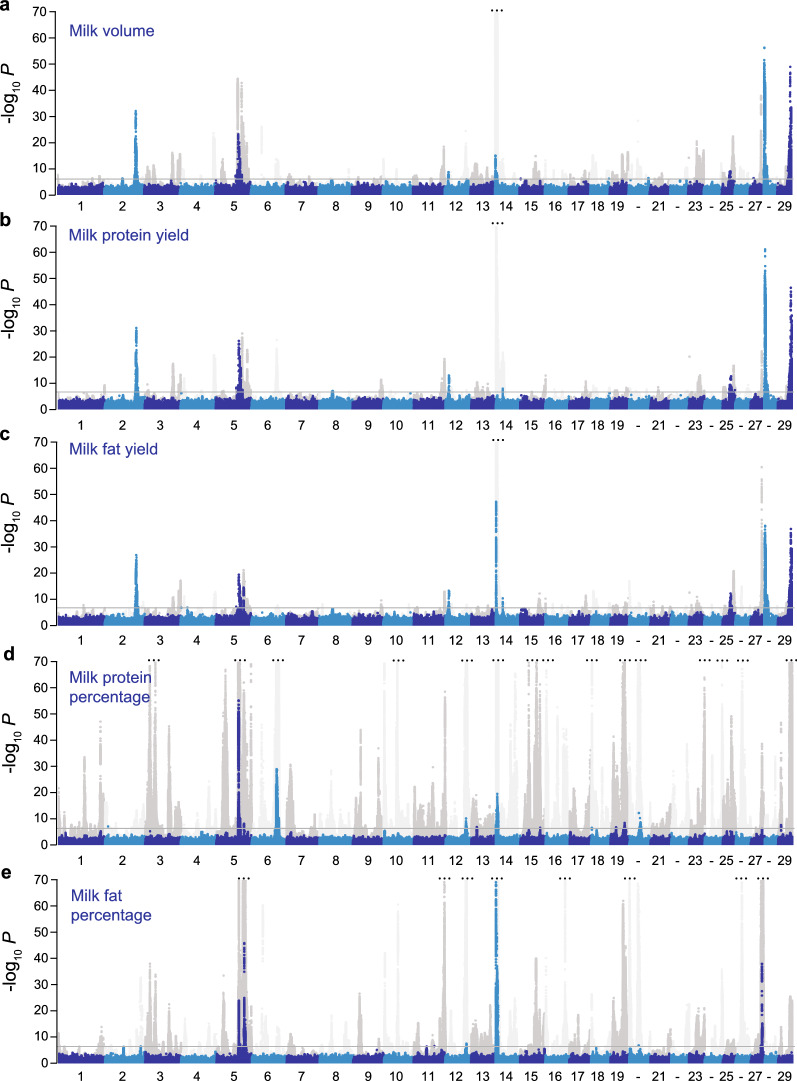
Dominance and additive Manhattan plots for lactation traits. Manhattan plots for milk volume (**a**), milk protein yield (**b**), milk fat yield (**c**), milk protein percentage (**d**), and milk fat percentage (**e**) showing significance of genotypic dominance (blue and light blue), and additive (grey and light grey) estimates for ~ 16.6 million imputed sequence variants. Chromosomes are differentiated by alternating colours and a grey line indicates the false discovery rate of 1 × 10^–3^, used to account for multiple testing. The y-axes are truncated for display purposes (indicated by 3 dots); chromosome numbers are shown on the x-axis (labels for chromosomes 20, 22, 24, 26 and 28 are not shown for clarity of display)

We performed GWAS across the five milk traits of interest, namely milk volume, milk protein yield, milk fat yield, milk protein percentage, and milk fat percentage, to identify non-additive QTL (Fig. 1). Both additive and dominance effects are included in these plots, and the iterative analysis identified 23 dominance QTL signals that were above the FDR threshold of 1 × 10^–3^. Some of the QTL were identified for multiple traits. These dominance QTL included 10, 11, 12, 8, and 7 QTL represented by 4618, 2706, 8525, 8987, and 5800 significant variants for milk volume, milk protein yield, milk fat yield, milk protein percentage, and milk fat percentage, respectively. The QTL spanned 13 discrete autosomes. After the iterative COJO analysis, standard additive GWAS identified 217, 152, 142, 673, and 457 QTL for milk volume, milk protein yield, milk fat yield, milk protein percentage, and milk fat percentage, respectively.

### Dominance QTL

**Fig. 2:**
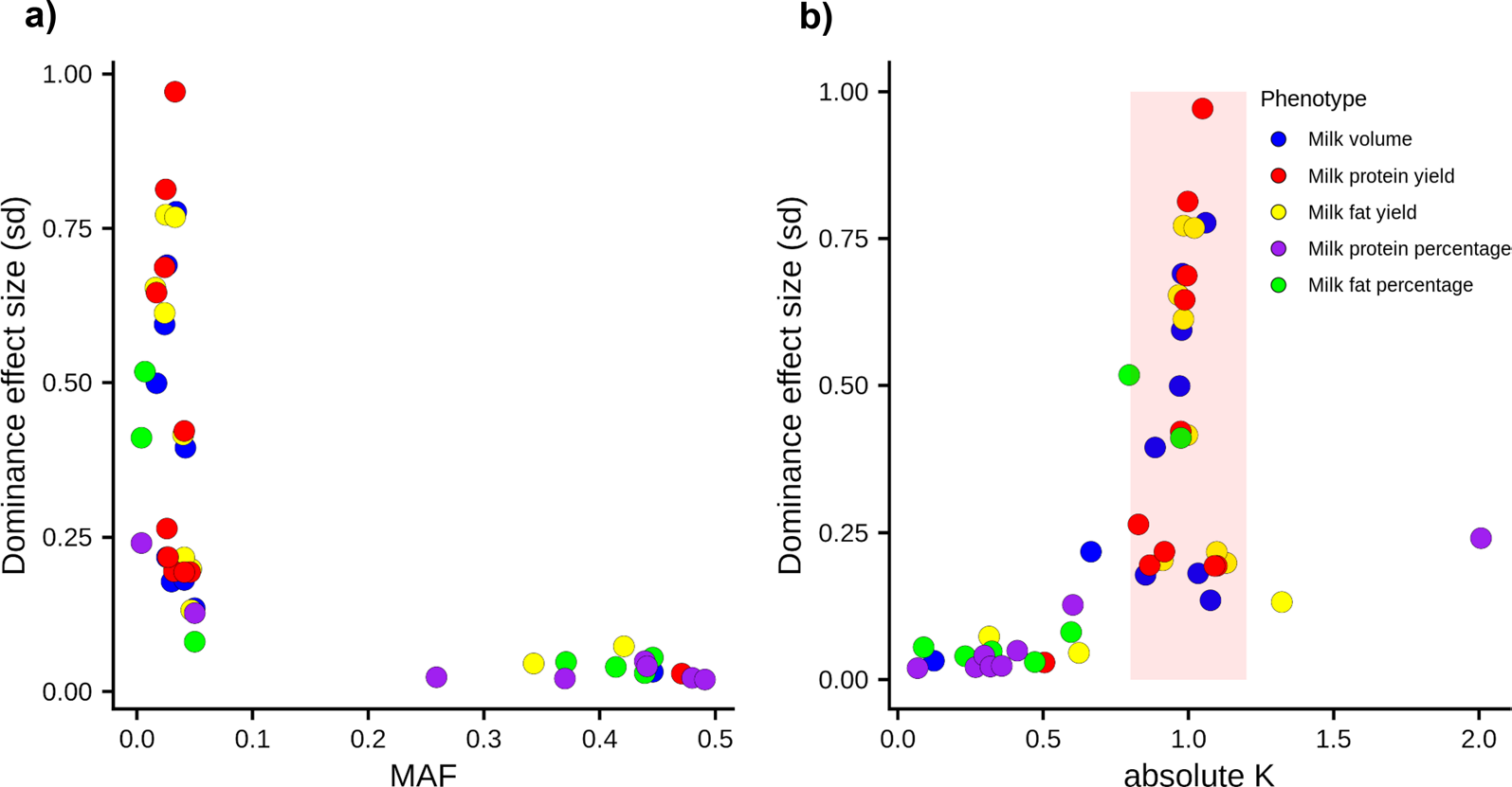
Plots presenting the genetic architecture of significant dominance QTL from GWAS on milk volume, milk protein yield, milk fat yield, milk protein percentage, and milk fat percentage. The plots contrast the minor allele frequency (MAF) against the dominance effect size (**a**), and the absolute value of k, where $k=d/|a|$, against the dominance effect size (**b**)

We identified 15 significant dominance QTL for milk yield traits, and 11 for milk composition traits (Table 2) and (see Additional file 1: Table S1). Twelve of the 15 milk yield dominance QTL had recessive effects and were located on chromosomes 2, 4, 5, 8, 12, 25, 28, or 29. Seven of these signals did not appear to have been previously reported, whereas the remainder were highlighted in our analysis [12] of growth and developmental traits in a population that overlapped with that described here. Eight of the 11 milk composition dominance QTL presented partial dominance effects, of which six were identified in our previously published additive GWAS (see Additional file 1: Table S1). Figure 2a compares the minor allele frequency and the size of the effect of the dominance components for all these loci. Interestingly, milk composition QTL appeared to be tagged by high minor allele frequency variants with comparatively small effects, whereas milk yield QTL were tagged by variants that had low minor allele frequencies and large effects. The type of effects also appeared to differ between traits (Fig. 2b), where milk yield traits were mostly impacted by recessive QTL, whereas milk composition traits near-exclusively presented QTL showing partial dominance.

**Table 2:** Association statistics for candidate mutations at recessive loci

Trait	QTL	Chr8_44 Mb	Chr25_24-27 Mb			Chr25_35 Mb	Chr27_15 Mb	Chr28_6-7 Mb
	Position	g.8.44119667T>A	g.25.24904939C>T	g.25.25161613G>A	g.25.26689392G>A	g.35975573C>T	g.27.15491451C>T	g.28.7922207G>A
	rsID	rs483207034	rs453138457	rs471945767	rs1116814780	NA	rs523126258	NA
	Candidate gene	*DOCK8*	*IL4R*	*KIAA0556*	*ITGAL*	*LRCH4*	*SLC25A4*	*RBM34*
	VEP	AA substitution	AA substitution	AA substitution	Premature stop	Premature stop	AA substitution	Premature stop
	Protein impact	p.His649Leu	p.Pro151Leu	p.Arg158His	p.Trp731*	p.Arg123*	p.Thr197Met	p.Arg55*
	SIFT score	0	0.02	0.14	NA	NA	0.01	NA
	MAF (HF/J/ALL)	0.013/0.059/0.03	0.001/0.043/0.017	0.001/0.042/0.016	0.002/0.049/0.019	0.034/0.001/0.031	0.046/0.001/0.027	0.044/0.004/0.043
Milk (L/lactation)	*a* ± sd	− 129.181 ± 23.604	− 218.249 ± 39.988	− 279.656 ± 49.108	− 169.491 ± 37.441	− 153.832 ± 24.201	− 123.607 ± 25.598	− 106.454 ± 17.786
	p	4.43E−08	4.82E−08	1.24E−08	5.99E−06	2.05E−10	1.38E−06	2.16E−09
	*d* ± sd	109.644 ± 23.905	215.668 ± 40.648	269.952 ± 49.887	161.062 ± 37.587	97.084 ± 245.537	120.056 ± 25.895	106.246 ± 17.929
	p	4.51E−06	1.12E−07	6.26E−08	1.83E−08	7.60E−05	3.55E+06	3.10E−09
	*k*	0.849	0.988	0.965	0.95	0.63	0.971	0.998
Fat (kg/lactation)	*a* ± sd	− 5.643 ± 1.177	− 11.827 ± 2.109	− 15.569 ± 2.359	− 9.708 ± 1.870	− 6.849 ± 1.137	− 7.075 ± 1.201	− 5.170 ± 0.866
	p	1.66E−06	2.05E−08	4.10E−11	2.09E−07	1.71E−09	3.84E−09	2.40E−09
	*d* ± sd	5.110 ± 1.181	11.339 ± 2.087	14.744 ± 2.372	9.022 ± 1.910	4.412 ± 1.133	5.729 ± 1.249	5.546 ± 0.859
	p	1.51E−05	5.56E−08	5.08E−10	2.33E−06	9.82E−05	4.48E−06	1.06E−10
	*k*	0.906	0.959	0.947	0.929	0.64	0.809	1.073
Protein (kg/lactation)	*a* ± sd	− 4.981 ± 0.870	− 9.226 ± 1.616	− 11.885 ± 1.834	− 7.847 ± 1.374	− 5.498 ± 0.838	− 5.008 ± 0.944	− 3.539 ± 0.587
	p	1.05E−08	1.12E−08	9.23E−11	1.11E−08	5.49E−11	1.14E−07	1.60E−09
	*d* ± sd	4.308 ± 0.897	9.023 ± 1.631	11.435 ± 1.829	7.497 ± 1.389	4.067 ± 0.844	4.595 ± 0.949	3.695 ± 0.592
	p	1.56E−06	3.14E−08	4.02E−10	6.77E−08	1.43E−06	1.30E−06	4.29E−10
	*k*	0.865	0.978	0.962	0.955	0.74	0.917	1.044

Linkage values with top variants are in Additional file 1: Table S1.

VEP: variant effect predictor; NA: not applicable or unknown; AA substitution: amino-acid substitution; *a*: genotypic additive effect; *d*: genotypic dominance effect; *k*: dominance coefficient; sd: standard deviation; p: p-value; MAF: minor allele frequency; HF: Holstein–Friesian; J: Jersey; ALL: all animals

### Identification of candidate causal mutations

**Fig. 3:**
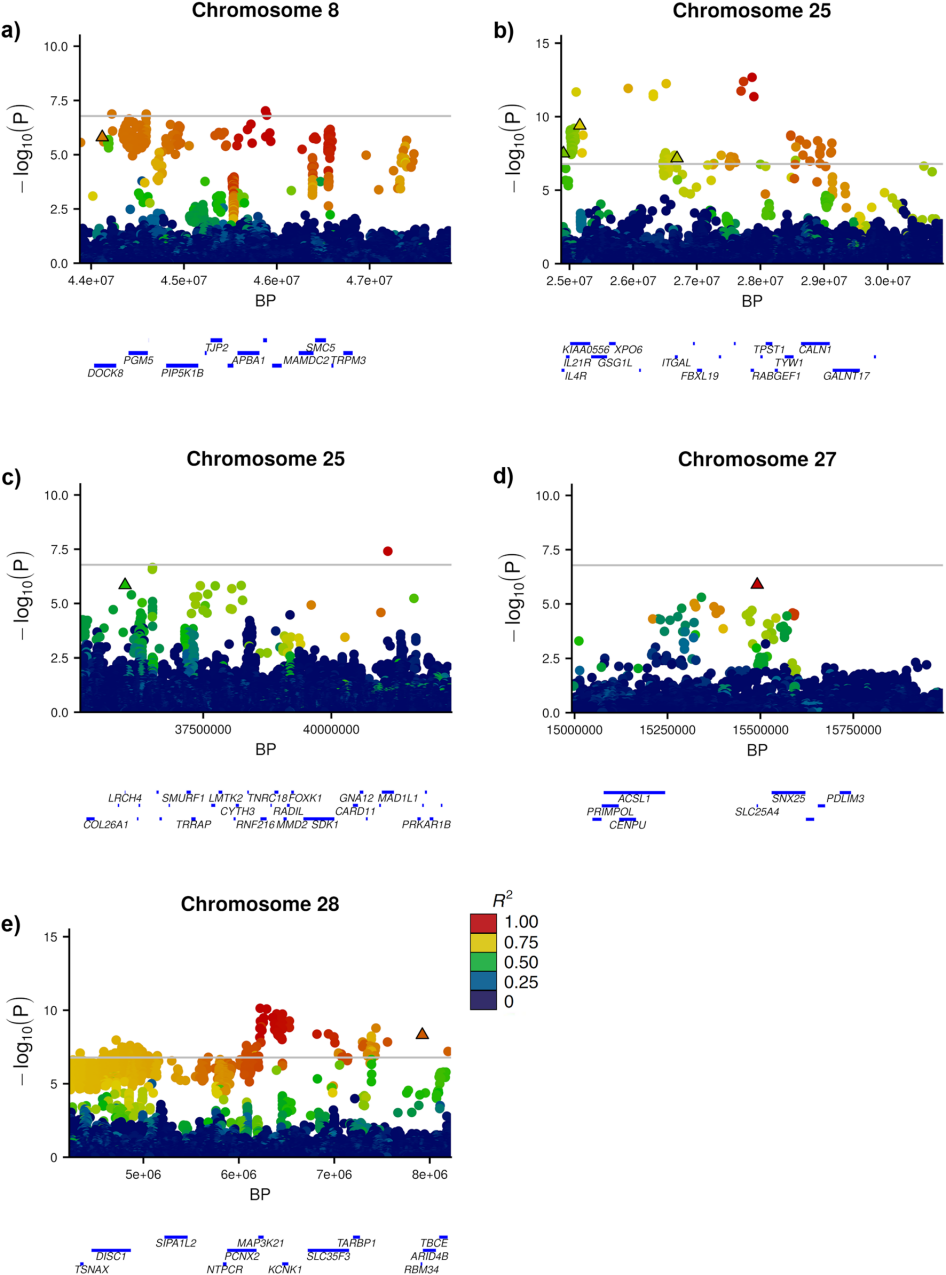
Manhattan plots for the five novel milk protein yield QTL representing the chr8:44Mbp (**a**), chr25:24-27Mbp (**b**), chr25:35Mbp (**c**), chr27:15Mbp (**d**), and chr28:7Mbp (**e**) loci. Variants are coloured by LD (R^2^) values with the top tag variant per locus, protein coding variants are shown as outlined triangles. Gene tracks are presented below each plot based on Ensembl 97, where gene names have been filtered on size

Given that the recessive milk yield QTL potentially represented novel bovine disorders, we prioritised these QTL for further investigation and selected those for which the dominance coefficient ($k$) was near 1 (0.7 < $k$ < 1.3). We used sequence annotations from VEP to highlight the mutations that might be responsible for these effects (Ensembl 97, [33]), i.e. pinpointing variants that were in strong to moderate LD (R^2^ > 0.7) with the lead variant *per locus*, and that were also predicted to alter or disrupt protein function. Furthermore, we manually investigated each QTL by visualising whole-genome sequence alignments that corresponded to animals with contrasting QTL genotypes. This step was performed to identify obvious structural mutations that were not detected by automated variant calling, i.e. those intersecting genes that could be similarly expected to modify or ablate gene function. However, we did not identify any structural variants that tagged QTL. It should be noted that these methods focussed only on protein-coding variants as candidates since, for recessive signals at least, we consider that protein altering mutations are primary candidates given the loss of function mechanism assumed to underlie recessive QTL. However, this does not preclude the involvement of regulatory variants, which we did not consider in our study. We identified five novel recessive QTL (including one near-significant recessive QTL), and several other previously identified recessive effects attributed to mutations in the *PLCD4*, *FGD4*, *MTRF1*, *GALNT2*, *DPF2*, and *MUS81* genes [12]. Figure 3 presents the position, regional LD, and association statistics for the QTL that are novel to this paper. Additional file 1: Table S1 shows all significant QTL identified, including those that are not described in detail here.

#### Chromosome 8

Chromosome 8 presented a significant signal at 45 Mb for milk protein yield and milk fat yield. The most significant variants for these signals (g.45878531A>C and g.45880948C>T) were in strong LD (R^2^ = 0.99), and an annotated missense variant (g.44119667T>A, rs483207034) was in high LD with both of the top-associated variants (R^2^ = 0.85 and 0.85, respectively; Fig. 3a). This variant in the *DOCK8* gene results in an amino acid (p.His649Leu) change and has a predicted deleterious impact (SIFT = 0).

#### Chromosome 25

A dispersed QTL signal was found on chromosome 25, spanning 24–27 Mb for the three lactation yield traits. The region presented different top-associated variants for milk fat yield (g.25921991AT>T) and milk protein yield and volume traits (g.27868969C>T). Variant effect prediction highlighted three candidate causal mutations in the region. These included a p.Pro151Leu substitution in the *IL4R* gene (g.24904939C>T, rs453138457) with R^2^ = 0.74, and 0.62, for the milk fat and milk protein/milk volume top variants, respectively, another missense variant (p.Arg158His) in the *KIAA0556* gene (g.25161613G>A, rs471945767) with R^2^ = 0.89, and 0.74, respectively, and a nonsense variant (p.Trp731*) in the *ITGAL* gene (g.26689392G>A, rs1116814780) with R^2^ = 0.76, and 0.70, respectively (Fig. 3b). Although all these variants represented plausible candidates to explain the QTL, we were not able to distinguish between the candidates through iterative analysis, since when any one of these candidates was fitted, the majority of the association for any of the other candidates was removed at this locus.

A second signal for protein yield on chromosome 25 was observed at 35 Mb. That locus maintained its significance after accounting for the QTL on chromosome 25 at 24–27 Mb through iterative analysis, suggesting that it was a different effect. The locus presented a strong candidate causative mutation that could underlie the effect, i.e. a stop gain mutation (g.35975573C>T; Arg123*) in the *LRCH4* gene that was the third most highly associated variant at this locus overall (Fig. 3c). We observed a mostly recessive effect for this variant ($k$ = 0.74), with the animals that carried the heterozygous and homozygous alternate genotypes producing 1.44 kg, and 11.21 kg less milk protein per lactation compared to the homozygous reference genotype. When g.35975573C>T was fitted as a fixed effect, the significance of the QTL was removed, and no other QTL was detected on the chromosome (see Additional file 2: Fig. S1).

#### Chromosome 27

We observed a signal at 15 Mb on chromosome 27 for milk protein yield. Although this did not exceed our q-value FDR threshold of 1 × 10^–3^ (equivalent to p-value = 1.65 × 10^–7^), this signal was notable given that the top variant (g.15491451C>T; rs523126258, p-value = 1.30 × 10^–6^) is a predicted deleterious missense mutation (p.Thr197Met) in the *SLC25A4* gene. Figure 3d shows a Manhattan plot for this region.

#### Chromosome 28

We previously reported a major recessive bodyweight QTL on chromosome 28 that corresponds to a likely causative splice acceptor mutation in the *GALNT2* gene (g.2281801G>A) [12]. This QTL was observed in the current analysis and impacted all three milk yield traits. However, iterative association analysis revealed a secondary QTL that is located approximately 4 Mb downstream of the *GALNT2* mutation at Chr28:6-7 Mb (top variant at g.6223350G>A). This residual signal highlighted a stop-gain non-sense mutation (g.7922207G>A) that is strongly linked to the g.6223350G>A variant (R^2^ = 0.89; Fig. 3e). This stop-gain mutation (p.Arg55*) is located in the *RBM34* gene, appears to be in linkage equilibrium with the *GALNT2* causal mutation (R^2^ < 0.001), and was not associated with bodyweight in our previous analysis (p = 0.37 [12]). A second GWAS iteration on chromosome 28 (fitting both *GALNT2* and *RBM34* mutations as fixed effects) did not reveal any other significant QTL on the chromosome (see Additional file 3: Fig. S2).

### Comparison between lactation and growth trait recessive QTL

We were interested in determining whether the novel recessive candidate causal mutations identified here had effects on the growth and developmental traits investigated in our previous study [12]. Here, we assessed the association statistics of these variants reported in that study, and while none of the novel mutations reached statistical significance (and would have thus been reported as part of that analysis), some did display apparent recessive mechanisms of moderate effect size. This suggests that, with increased sample sizes, these variants may present significant effects on growth traits. Notably, the mutation in *KIAA0556* was one of the most strongly associated variants for body condition score in that study, presenting the 10th smallest dominance p-value of the ~ 16 million variants tested in that analysis. Additional file 1: Table S2 includes the association statistics for five of the seven candidate causal mutations presented above (the *ITGAL* and *SLC25A4* mutations were not captured in the genotype dataset reported by Reynolds et al. [12]). All of the novel candidate mutations highlighted in Reynolds et al. [12] were also associated with lactation traits (see Additional file 1: Table S1) except for the *MYH1*-disrupting structural variant which was only associated with body condition score in that study.

### Dominance QTL for composition traits

In addition to the recessive QTL identified for milk yield traits, we also identified dominance QTL for milk composition traits. We investigated these effects and observed several partial dominance QTL that are in close proximity to previously described additive loci. The tag variants of these QTL were adjacent to the following genes: *CSF2RB* [37], *MGST1* [17], *DGAT1* [13], *GHR* [14], *GPAT4* [16], and *PICALM* [38] and, in each case, these variants were in high LD (R^2^ > 0.8) with previously identified causal and/or tag variants (see Additional file 1: Table S1).

Milk protein percentage presented multiple dominance QTL on chromosome 6 within the 80 to 85 Mb region (see Additional file 1: Table S1). Among these QTL, the most significant variant (g.84112451C>A) showed a partial dominance effect. Unlike in the above examples, we did not identify any very strongly linked candidate mutation although this variant was in moderate LD with a previously proposed causative variant in the *CSN1S1* gene (R^2^ = 0.53; p.Glu192Gly mutation; g.85427427A>G) [39]. Chromosome 12 presented a significant dominance QTL, for which we observed a partial dominance effect at 68 Mb for milk protein percentage with the top variant at g.68763031T>TG. As observed for the chromosome 6 locus, no particularly obvious candidate causal variant or gene was identified that might account for that signal.

### Comparison between the additive and dominance GWAS results

**Fig. 4:**
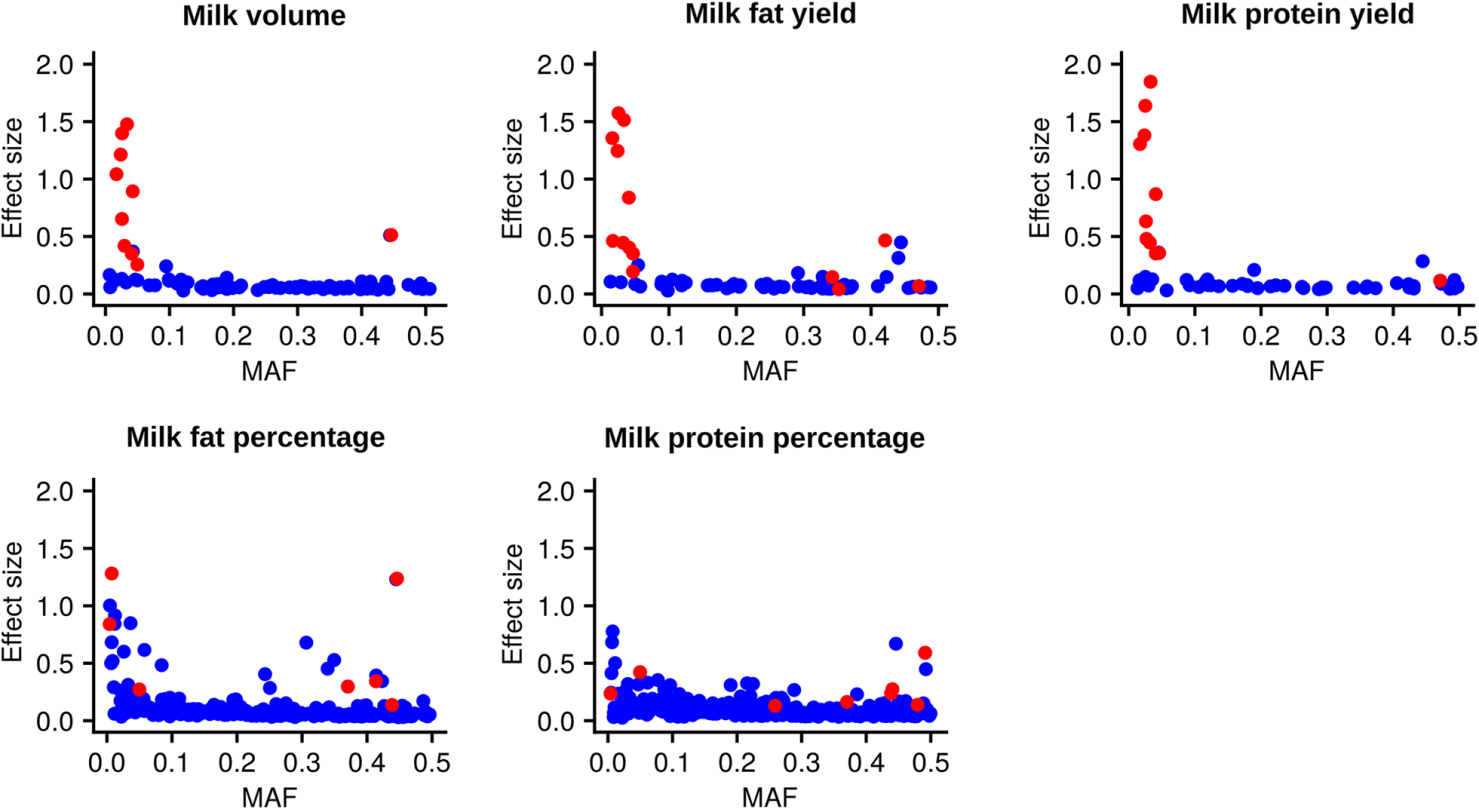
Plots contrasting minor allele frequency (MAF) and the absolute effect size between homozygote genotype classes (effect size) for additive (blue) and dominance (red) QTL detected via GWAS across lactation traits

Figure 4 compares the minor allele frequency (MAF) and the effect sizes between homozygous genotypes across all traits and genetic mechanisms. As expected, we observed many more additive QTL than dominance QTL across all traits. On the one hand, it is noteworthy that the mutations detected via dominance GWAS for milk yield traits had very large effects compared to the additive QTL detected for these traits, and most of them had a recessive effect. On the other hand, the largest effects observed for the two milk composition traits were mostly additive QTL, and dominance effects tended to have high MAF and presented mostly partial dominance effects.

## Discussion

Our results highlight the presence of many non-additive QTL for milk traits in cattle. The majority of these signals for milk yield traits present recessive QTL, that involve five novel loci and several previously described recessive loci [12]. Although the milk protein percentage and milk fat percentage traits also yielded many dominance GWAS signals, most of them correspond to partially dominant effects that are attributable to previously reported additive QTL.

### Different trait classes present contrasting additive and non-additive genetic architectures

One remarkable observation from our study is the apparent difference in additive and non-additive genetic architectures between milk yield traits and milk composition traits. Dominance heritabilities for the milk yield traits ranged from 3 to 7%, whereas for the milk composition traits they were zero or near zero. In contrast, the additive heritabilities ranged from 23 to 31% for the milk yield traits and from 64 to 70% for the milk composition traits. These findings are consistent with those of Sun et al*.* [9] who report similar additive and dominance heritabilities, and suggest that dominance, in particular recessive mechanisms, may play a bigger role in the regulation of milk yield traits than that of composition traits.

These differences in the genetic architecture of the milk traits investigated in this study were also observed when the properties of individual dominance QTL were compared between milk yield and milk composition traits. The majority of the dominance QTL identified for milk yield traits had recessive genetic effects, while the majority of the milk composition traits had partial dominance effects. Furthermore, the dominance QTL for milk yield traits were characterised by low MAF and large effect sizes, whereas those for milk composition traits were characterised by high MAF and comparatively smaller effect sizes. We hypothesize that these observations reflect the way in which different traits may represent underlying recessive syndromes—i.e., their utility as proxies for genetic disorders. Among all the recessive QTL detected in our study, a subset of these had previously been validated as representing new genetic disorders [12]. Although we did not investigate the novel recessive loci in this study with the same rigour as those analysed in Reynolds et al. [12], their very large, uniformly negative effects suggest that at least some of them will be similarly validated. Notably, none of these loci (new or old) show substantial effects on milk composition, suggesting that milk fat and protein percentage traits do not readily reflect recessive effects. This finding can be rationalised by the comparatively broad range of biological processes expected to impact milk yield traits (or the growth and development traits investigated in Reynolds et al*.* [12]), where the energy demands of lactation (or growth) may manifest a wide range of other organismal stresses. In contrast, the relative composition of milk components likely represents a narrower spectrum of mammary-specific biological mechanisms, and thus we hypothesise that these traits are less able to serve as proxies of animal fitness.

It should be acknowledged that given that protein yield and fat yield are the products of milk volume and their respective percentages, these traits are not independent. We observed that the variance components and the genetic architectures of milk fat yield and milk protein yield are more comparable to milk volume than their respective composition traits.

### Previous studies highlighting recessive effects on quantitative traits

As discussed above, we recently reported an investigation of growth and developmental traits that identified non-additive QTL using similar approaches to those presented here [12]. That study demonstrated how quantitative traits can be used as proxies to map genetic disorders without prior disease identification. In doing so, we highlighted several recessive QTL represented by variants in the *PLCD4*, *FGD4*, *MTRF1*, *GALNT2*, *DPF2*, and *MUS81* genes, each with large effects on bodyweight and other quantitative traits. The work presented in the current paper builds on those findings; we identified many of the same recessive mutations as well as several additional recessive QTL. Some of these additional QTL displayed moderate but not significant recessive effects for growth traits and their discovery may be assumed to reflect the increased sample sizes leveraged in the current study. These findings suggest that milk yield traits might also be used to represent whole-animal health, and since lactation measurements are more routinely derived than bodyweight phenotypes (at least in bovine dairy systems), these likely represent a more accessible phenotype relevant to a larger number of international evaluation systems.

Few studies other than Reynolds et al. [12] have highlighted major recessive effects using quantitative trait data. Although non-additive GWAS with large sample sizes have been performed in cattle [11, 36], the low density of the SNP arrays used in those earlier studies may have hampered the ability to directly resolve candidate causative variants [12]. This challenge arises due to the different LD properties between causal and observed variants for additive and non-additive QTL, such that the variance that an observed variant can explain decreases by R^2^ for additive QTL, and by R^4^ for dominant or recessive QTL. This means that the observed tag variants need to be more closely linked to the causal dominance variants to capture the QTL [40, 41]. The fact that major deleterious alleles are also likely to be infrequent compounds this problem. Under Hardy–Weinberg expectations where p^2^ + 2pq + q^2^ = 1, the number of rare allele homozygotes (q^2^) decreases exponentially as allele frequency decreases. Practically, this means very large sample sizes are needed to represent rare allele homozygotes, where at 1% MAF, 10,000 individuals would be expected to present a single homozygote (with 1,000,000 individuals required at MAF = 0.1%). However, as sample sizes and high-density genotyping platforms begin to permit such analyses, we anticipate similar such studies in other populations to begin to appear. One recent, noteworthy such study has suggested the importance of recessive variants in the context of male fertility and semen traits in cattle [42]. In that study, recessive QTL and candidate causal mutations were identified in several genes including a missense variant in the *SPATA16* gene. That discovery was based on imputed genotypes at high density (the Illumina BovineHD platform), but the size of the studied population was quite small (N = 3736 bulls). It is likely that the discovery of these QTL was partly aided by the remarkable frequency of the deleterious haplotypes identified in that study, presenting allele frequencies ranging from 9 to 34% [42].

### Recessive QTL of interest

Although many non-additive signals were identified in our study, we were particularly interested in the recessive QTL with large effects, given that these might represent underlying genetic disorders. We highlighted protein-coding variants as candidates because we considered these to be the most probable causal variants, but we acknowledge this is a relatively simple approach and that regulatory or unidentified structural variants may alternatively underlie these recessive QTL. These caveats aside, the five novel recessive QTL on chromosomes 8, 25, 27, and 28 are presented and discussed below.

#### Chromosome 8—*DOCK8*

Our results present a missense mutation in the *DOCK8* gene as potentially having a deleterious recessive impact on milk yield traits. The QTL appears to operate in a completely recessive manner, with the *DOCK8* variant present at low allele frequencies in each breed (Holstein–Friesian MAF = 0.013 and Jersey MAF = 0.059). The *DOCK8* gene encodes dedicator of cytokinesis 8, a member of the DOCK180 family of guanine nucleotide exchange factors, which influences intracellular signalling networks and is important in immune responses and lymphocyte regulation in humans and mice [43]. Recessive mutations in *DOCK8* have been associated with the hyper immunoglobulin E syndrome which leads to the onset of an immunodeficiency disease combined with other health complications [44]. In mice, compromised immune responses are also observed including negative impacts on B cell migration [45], and T cell migration and viability [46, 47]. *DOCK8* variants have not previously been associated with cattle performance traits, but if this missense mutation underlies the QTL on chromosome 8, we hypothesized that it could act through similar negative impacts on the immune system. Under this hypothesis, it is unknown whether the effects on lactation are due to mammary immune function or secondary impacts. However, given that higher levels of circulating immunoglobulins E and lymphocyte profiling can indicate *DOCK8* deficiency in humans [44, 48], it would be interesting to sample and profile homozygous animals to definitively establish the causality of the *DOCK8* missense mutation for this QTL.

#### Chromosome 25—*IL4R*, *KIAA055*6, *ITGAL*

The QTL identified on chromosome 25 at 24–27 Mb presented three candidate mutations in the *IL4R*, *KIAA0556*, and *ITGAL* genes. The *IL4R* gene encodes the interleukin 4 receptor, which is a transmembrane protein involved in immune responses in humans [49]. The *KIAA0556* gene is associated with microtubule regulation in humans, and *KIAA0556* knockout mutations in humans and mice have been associated with Joubert syndrome, a neurological disorder [50]. The *ITGAL* gene encodes the integrin alpha L chain, and loss of function variants in this gene have been associated with compromised immunity including increased susceptibility to infection to Salmonella in mice [51]. Given that the iterative association analysis failed to prioritise one of these variants over the other, it is unknown which of these variants might be responsible for the QTL, and our focus on protein-coding variants as candidates may have also overlooked alternative non-coding or structural mutations. These variants are nevertheless in moderately strong, though not in perfect LD (maximum pairwise R^2^ = 0.79), thus physical genotyping for fine mapping and future functional testing should help to resolve the identity of the gene (or genes) underpinning this QTL.

#### Chromosome 25—*LRCH4*

Although iterative GWAS did not resolve candidates in the above example, this approach did highlight a second QTL on chromosome 25 represented by a nonsense mutation in the *LRCH4* gene, which encodes leucine-rich repeats and calponin homology containing protein 4. It regulates the signalling of toll-like receptors (TLR) and has been shown to influence innate immune responses in mice [52]. In that study, researchers showed that *LRCH4*-silenced cells presented a reduced expression across pro-inflammatory cytokines produced in the TLR4 pathway, most notably in that of IL-10 and MCP-1. We hypothesise that the *LRCH4* knockout mutation identified in our study may have negative impacts on the innate immunity of cattle, and that those impacts could lead to the recessive effects we observed on milk volume, milk fat yield, and milk protein yield.

#### Chromosome 27—*SLC25A4*

While non-significant at the genome-wide level (cf. p = 1.65 × 10^–7^ vs p = 1.30 × 10^–6^), the locus on chromosome 27 at 15.5 Mb presented a conserved amino acid mutation in the *SLC25A4* gene as the lead associated variant and was therefore of interest. This variant demonstrated a complete recessive effect on all three lactation yield traits. The *SLC25A4* (*solute carrier family 25 member 4*) gene encodes the adenine nucleotide translocator (Ant1) protein, responsible for the translocation of ATP and ADP between the cytoplasm and mitochondria. In mice, *SLC25A4* knockouts result in mitochondrial myopathy and cardiomyopathy, and severe intolerance to exercise [53]. Similarly, in humans, childhood-onset mitochondrial disease and exercise intolerance have been observed for both dominant [54] and recessive mutations [55] in *SLC25A4*. Given the implication that mitochondrial functional deficits might underlie the negative lactation effects highlighted in the current study, it would be intriguing to examine the phenotypes of homozygous cows further in this context.

#### Chromosome 28—*RBM34*

At first glance, the strong associations with the lactation yield traits on chromosome 28 might reasonably be attributed to the previously reported splice site mutation in *GALNT2* [12]. However, when this mutation was fitted as a covariate in our iterative GWAS, a secondary signal was observed, highlighting a nonsense mutation in the *RBM34* gene as potentially responsible for the effect. The *RBM34* gene encodes an RNA recognition motif protein with an RNA-binding domain. The literature on *RBM34* in humans or model organisms is scarce, with limited implication of the gene in embryonic stem cell differentiation [56]. Here, we observed a predicted homozygous knockout of *RBM34* that may influence milk volume, milk protein yield, and milk fat yield in a recessive manner, although its status as a largely uncharacterised RNA-binding protein leaves little room for speculation as to how these effects might manifest. Mechanism aside, the identification of two co-locating, yet uncorrelated recessive QTL demonstrates the utility of using iterative GWAS approaches, given that conventional analysis would likely fail to differentiate these effects. We note that other researchers have observed effects on lactation at the 6–10 Mb locus [57]. However, the LD (R^2^ with *RBM34* = 0.04, *GALNT2* = 0.02) between the tag variant identified by Raven et al*.* [57] (rs41607517) and the nonsense mutations identified here is very low, which suggests that they are different effects.

### Previously described additive QTL present partial dominance

We observed several partial dominance QTL that are closely linked to previously described QTL identified from standard additive analyses. As presented in Additional file 1: Table S1 we identified dominance components in high LD with variants associated with the *CSF2RB* [37], *MGST1* [17], *DGAT1* [13], *GHR* [14], *AGPAT6* [16], *PLAG1* [58, 59], and *PICALM* [38] genes (and in moderate LD with a variant in the *CSN1S1* gene [39]). These partial dominance associations were mostly identified in milk composition traits. These observations suggest that many well-known major-effect QTL that are identified in additive GWAS’ incorporate some level of non-additivity, in agreement with the analyses of milk traits reported by Jiang et al. [11, 36].

## Conclusions

In this study, we have highlighted that different classes of lactation traits (yield compared to composition traits) present different additive and non-additive genetic architectures. We speculate, that these differences derive from dissimilarities in the cellular and molecular manifestation of these traits, and although milk yield traits have comparatively low additive heritabilities, these traits may better reflect whole-animal energy and fitness status and be a better proxy of a wider range of underlying biological disorders. At the single locus level, we identified five QTL presenting seven candidate causative variants in the *DOCK8*, *IL4R*, *KIAA0556*, *ITGAL*, *LRCH4*, *SLC25A4*, and *RBM34* genes, highlighting medium- to large-effect recessive variants that may provide future opportunity for diagnostic testing and animal improvement.

## Supplementary Information

**Additional file 1: Table S1.** Association statistics for most significant variants. Table including association statistics for the most highly associated variant for each QTL identified through GWAS. **Table S2.** Association statistics for growth and developmental traits. Table including association statistics for five candidate causal mutations in the *DOCK8*, *IL4R*, *KIAA0556*, *LRCH4*, and *RBM34* genes for growth and developmental traits [12].

**Additional file 2: Figure S1.** Iterative Manhattan plots for milk-protein yield on chromosome 25. Blue indicates the candidate causal variants in genes; *IL4R*, *KIAA0556*, and *ITGAL*, and red indicates the candidate causal variant in the *LRCH4* gene. A grey line indicates the false discovery rate of 1 × 10^–3^, used to account for multiple testing.

**Additional file 3: Figure S2.** Iterative Manhattan plots for milk-protein yield on chromosome 28. Blue indicates the candidate causal variant in the *GALNT2* gene, and red indicates the candidate causal variant in the *RBM34* gene. A grey line indicates the false discovery rate of 1 × 10^–3^, used to account for multiple testing.

## Data Availability

A subset of whole-genome sequences used for imputation of the genotypes presented in this paper have been deposited in the SRA database [60]. Additional data is available on reasonable request with the permission of Livestock Improvement Corporation, contingent on the execution of an appropriate transfer agreement.
