# Supplementary figures and images for: Non-additive QTL mapping of lactation traits in 124,000 cattle reveals novel recessive loci
Source: Genet Sel Evol. 2022 Jan 24;54:5. doi: 10.1186/s12711-021-00694-3 (PMC8785530; doi:10.1186/s12711-021-00694-3)

Iteration 0

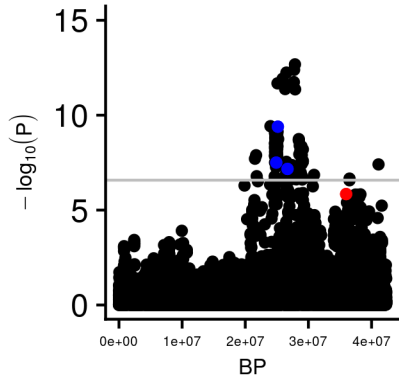

Iteration 1

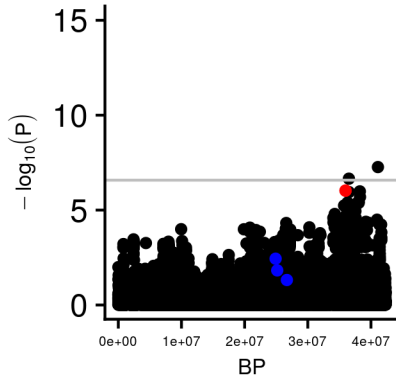

Iteration 2

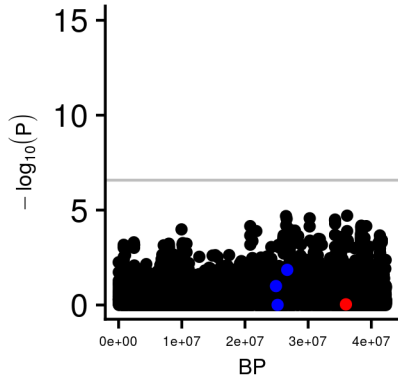

Supplement: Supplementary file 2 — Additional file 2: Figure S1. Iterative Manhattan plots for milk-protein yield on chromosome 25. Blue indicates the candidate causal variants in genes; IL4R, KIAA0556, and ITGAL, and red indicates the candidate causal variant in the LRCH4 gene. A grey line indicates the false discovery rate of 1 × 10^–3^, used to account for multiple testing. [file 12711_2021_694_MOESM2_ESM.pdf]

Iteration 0

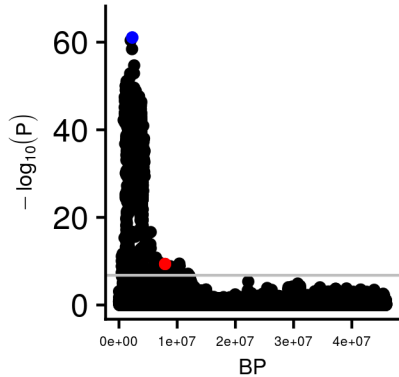

Iteration 1

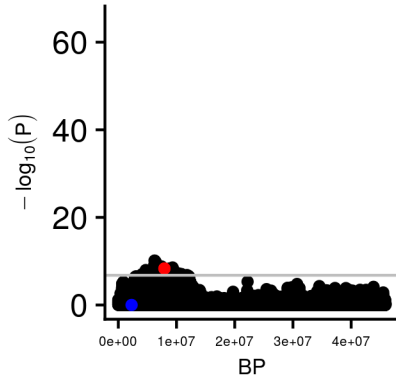

Iteration 2

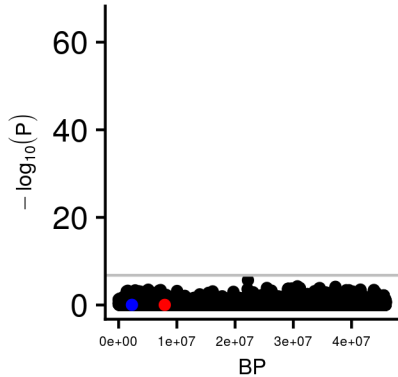

Supplement: Supplementary file 3 — Additional file 3: Figure S2. Iterative Manhattan plots for milk-protein yield on chromosome 28. Blue indicates the candidate causal variant in the GALNT2 gene, and red indicates the candidate causal variant in the RBM34 gene. A grey line indicates the false discovery rate of 1 × 10^–3^, used to account for multiple testing. [file 12711_2021_694_MOESM3_ESM.pdf]
